# Interrogating the Transient Selectivity of Bacterial Chemotaxis-Driven Affinity and Accumulation of Carbonaceous Substances *via* Raman Microspectroscopy

**DOI:** 10.3389/fmicb.2019.02215

**Published:** 2019-10-04

**Authors:** Hanbing Li, Francis L. Martin, Kevin C. Jones, Dayi Zhang

**Affiliations:** ^1^State Key Laboratory of Pollution Control and Resource Reuse, School of the Environment, Nanjing University, Nanjing, China; ^2^Lancaster Environment Centre, Lancaster University, Lancaster, United Kingdom; ^3^School of Pharmacy and Biomedical Sciences, University of Central Lancashire, Preston, United Kingdom; ^4^School of Environment, Tsinghua University, Beijing, China

**Keywords:** *Acinetobacter baylyi*, *Pseudomonas fluorescence*, *Escherichia coli*, chemotaxis, transient selectivity, Raman microspectroscopy

## Abstract

Carbonaceous substances are fundamental organic nutrients for microbial metabolism and catabolism in natural habitats. Microbial abilities to sense, accumulate, and utilize organic carbonaceous substances in the complex nutrient environment are important for their growth and ecological functions. Bacterial chemotaxis is an effective mechanism for microbial utilization of carbonaceous substances under nutrient depletion conditions. Although bacterial accumulation and utilization to individual carbonaceous substance in long-term cultivation has been well studied, their selective affinity of mixed carbonaceous substances remains to be investigated, primarily because of technical limitations of conventional methods. Herein, we applied Raman microspectroscopy to identify chemotaxis-driven affinity and accumulation of four organic carbonaceous substances (glucose, succinate, acetate, and salicylate) by three bacterial strains (*Acinetobacter baylyi*, *Pseudomonas fluorescence*, and *Escherichia coli*). *A. baylyi* exhibited strong binding affinity toward glucose and succinate, whereas *P. fluorescence* and *E. coli* were preferentially responsive to glucose and acetate. For the first time, bacterial transient selectivity of carbonaceous substances was studied *via* interrogating Raman spectral alterations. Post-exposure to carbonaceous-substance mixtures, the three bacterial strains showed distinct selective behaviors. Stronger selective affinity enhanced the chemotaxis-related signal transduction in *A. baylyi* cells, whereas the carbonaceous substance signal transduction in *E. coli* was decreased by higher selective affinity. In *P. fluorescence*, there was no specific effect of selective affinity on signal transduction. Our study suggests that Raman microspectroscopy can successfully investigate and distinguish different scenarios of bacterial competitive and transient unitization of organic carbonaceous substances.

## Introduction

Bacteria can employ diverse mechanisms to sense and accumulate chemical molecules in their natural environment (Adler, 1966; Sourjik and Wingreen, 2012). The bacterial chemotaxis system is a well-developed chemical gradient sensor, and motile chemotactic bacterial cells are able to follow concentration gradients of chemo-attractants, such as nutrients and other environmental stimuli, to select and accumulate them on cell membrane (Sourjik and Wingreen, 2012). Attractant nutrients include organic carbonaceous substances, organic nitrogen and other essential inorganic elements. Organic carbonaceous substances, e.g., sugar, organic acids and polyols, are fundamental nutrients for bacterial growth. Bacteria show positive and selective responses toward some specific carbonaceous substance molecules present in medium (Adler, 1966; Singh and Arora, 2001; Sourjik, 2004; Kirby, 2009; Sampedro et al., 2015). Glucose is a major carbonaceous substance exhibiting high attraction to microbes (Hazelbauer et al., 1969; Singh and Arora, 2001; Weert et al., 2002), and carbonaceous acids such as succinate and acetate can be used as chemo-attractant in bacterial chemotaxis assays (Dailey and Berg, 1993; Oku et al., 2014; Sampedro et al., 2015). Bacterial chemotaxis refers to the movement of cells toward attractants or repellents (Sampedro et al., 2015). Favorable attractants of carbonaceous acids lead to continuous movement promoting their further accumulation and utilization in bacterial cells (Sly et al., 1993). Microbial chemotaxis-related affinity toward carbonaceous substances is vital for their competitive advantages in natural habitats as it assists microbes in accessing carbonaceous nutrients, and determines the accumulation and utilization of carbonaceous nutrients in bacterial cells.

Bacterial chemotactic responses to carbonaceous substances are regulated by specific proteins. In *Pseudomonas*, methyl-accepting proteins (M) are believed to determine the specificity of the chemotactic response (Ferrández et al., 2002). MCP comprises the periplasmic ligand-binding region and the cytosolic methyl-accepting domain that allows sense and transfer of the chemical signal to bacterial cells (Ferrández et al., 2002; Alexander and Zhulin, 2007). The chemotaxis protein McpS in *Pseudomonas putida* KT2440 is the receptor-sensing tricarboxylic acid (TCA) cycle intermediate (Lacal et al., 2011), and the glucose-specific binding protein GLTB is characterized in *P. aeruginosa* (Sly et al., 1993). *E. coli* is a model strain for chemotaxis study (Sourjik, 2004; Sourjik and Wingreen, 2012), in which the CheA and CheW combine as a ternary complex to facilitate chemotactic responses to carbonaceous substances such as glucose and acetate (Maddock and Shapiro, 1993; Sourjik and Berg, 2000). It is reported that *mcp*, *cheR*, and *chew-2* genes in *Acinetobacter* are responsible for chemoreceptor cluster formation (Wang and Shao, 2014). The flagella on the *Acinetobacter* cell membrane facilitate the movement toward carbonaceous molecules (Foster, 1962). However, mechanisms of glucose or succinate chemotaxis in *Acinetobacter* remain unknown.

Approaches used to study bacterial chemotaxis include swarm plates (Henrichsen, 1972), capillary assays (Berg and Turner, 1990), temporal stimulation of tethered cells (Block et al., 1983), and automated tracking of swimming cells (Shi et al., 2006; Kearns, 2010). These approaches are versatile in testing bacterial chemotaxis toward specific individual chemicals. They can successfully describe the movement of bacterial cells to a certain carbonaceous substance; however, they fail in distinguishing which molecule is chemotactically sensed and accumulated by bacterial cells in a mixture of substances. In contrast, Raman microspectroscopy can provide the vibrational information on chemicals acquired by bacteria (Li et al., 2017). It is a fast, reproducible and non-destructive approach, and widely applied in biological studies (Efrima and Zeiri, 2009). The effects of the co-existence of carbonaceous substances on bacterial sensing and accumulation require further investigation, because it represents a real-world scenario of carbonaceous substance utilization by bacteria and explains the microbial ecological functions in complicated natural habitats.

Well-developed to determine cell-molecule interactions and the microbial response to the environment (Efrima and Bronk, 1998; Guzelian et al., 2002; Wang et al., 2011; Zhou et al., 2015; Jin et al., 2017b), Raman microspectroscopy has been applied to investigate nanotoxicity in bacterial cells (Cui et al., 2013) and to determine the minimum inhibitory concentrations of antibiotics using heavy water labeling (Tao et al., 2017). Recently, the alkane chemotactic affinity of *A. baylyi* is studied *via* Raman spectral alterations to illustrate the intracellular bioaccumulation in these cells (Li et al., 2017). Although it is well-documented that Raman spectroscopy is sophisticated in discriminating spectral alterations of bacterial cells after long-term cultivation with different carbon sources (Maquelin et al., 2000; Schuster et al., 2000), the study of bacterial transient affinity and uptake of diverse carbonaceous substances *via* Raman spectra remains covered. The fast manners of biospectroscopy allow the measurement of binding affinity and accumulation of molecules on/inside bacterial cells, hinting at the possibility of characterizing the fingerprints of carbonaceous substances bioaccumulated by bacteria and to distinguish their transient and selective responses. To our knowledge, there is no such study exploring Raman spectral alterations in bacterial transient affinity toward and selective uptake from a mixture of organic carbonaceous substances.

Based on our recently developed Raman spectroscopic method for determining chemotactic affinity and accumulation of alkane molecules in *A. baylyi* cells (Li et al., 2017), this work further distinguishes the Raman spectral alterations and quantifies the bacterial transient affinity and accumulation of hydrophilic carbonaceous substances (glucose, succinate, acetate, and salicylate) after short-time exposure (30 min). By interrogating the selective affinity and accumulation in *Acinetobacter baylyi*, *Pseudomonas fluorescence*, and *Escherichia coli* toward individual carbonaceous substance and their mixtures *via* Raman spectra, we found the selective behavior of bacterial cells after transient exposure to carbonaceous substance mixtures. Additionally, Raman spectral alterations revealed the distinct mechanisms of chemotaxis-related signal transduction in the three bacterial strains. Signal transduction was enhanced or weakened by attractant binding affinity in *A. baylyi* or *E. coli*, respectively, whereas no significant effect of attractant binding affinity on the signal transduction was observed for *P. fluorescence*. Our results provide a novel, rapid and cost-effective Raman spectroscopic technique in distinguishing bacterial selective affinity and accumulation of various nutrients *in situ*, potentially feasible in characterizing bacterial metabolic and catabolic behavior in their natural habitats.

## Materials and Methods

### Bacterial Strains and Growth Conditions

The bacterial strains used in this study included *Acinetobacter baylyi* ADP1, *Pseudomonas fluorescence*, and *Escherichia coli* JM109 (Jin et al., 2017a). They were grown in minimal medium (MM) with 20 mM sodium succinate as the sole carbon source, shaking at 150 rpm and 30°C for 16 h. To prepare mineral medium, 1.0 g of (NH_4_)_2_SO_4_, 2.5 g of KH_2_PO_4_, 0.1 g of MgSO_4_⋅7H_2_O, 0.005 g of FeSO_4_⋅7H_2_O, 0.25 g of nitrilotriacetic acid, 0.55 g of NaOH and 1 mL of Bauchop and Elsden solution were mixed well in 1.0 L deionized water and autoclaved (Jia et al., 2016). Bacterial cells were harvested by centrifugation at 4,000 rpm for 4 min and washed three times using sterile deionized water. Finally, the cell pellets were separately suspended in MM without any carbon source for further experiments. Unless specified otherwise, all chemicals used in this study were of analytical grade and purchased from Sigma-Aldrich (United Kingdom).

### Exposure to Carbonaceous Substances

The four studied carbonaceous substances included three organic acid salts (sodium succinate, sodium acetate, and sodium salicylate) and one carbohydrate (glucose). They were dissolved in MM to prepare the 1 M stock solution required. Bacterial suspensions were mixed with each carbonaceous substance stock solution to obtain a final concentration of 10 mM. The transient exposure was 30 min at 30°C for all treatments. From our previous study, all the three strains stayed in the lag phase for at least 2 h after re-cultivated in MM with different carbon sources and exhibited distinct spectral fingerprints comparing to those after the long-term cultivation (usually 12–16 h) (Jin et al., 2018). Their Raman spectral alterations, after transient exposure, were therefore entirely different from the alterations of bacterial cells after long-term cultivation with different carbon sources. Bacterial suspensions post-exposure to carbonaceous substances were centrifuged at 5,000 rpm for 5 min to remove the medium, and the cell pellets were washed once with sterile deionized water prior to Raman spectral acquisition.

The dose response of carbonaceous substance binding affinity and accumulation was measured by exposing bacterial suspensions to the carbonaceous substance with high binding affinity: succinate/glucose for *A. baylyi* and acetate/glucose for *P. fluorescence* and *E. coli*. After adding different volumes of carbonaceous substance stock solution to 1 mL bacterial suspensions to reach the final concentrations of 0, 1, 5, and 10 mM, the mixture was incubated for 30 min at 30°C, centrifuged at 5,000 rpm for 5 min and washed once with sterile deionized water prior to Raman spectra acquisition. Thirty-minute incubation at 30°C is the optimal exposure time for the most significant alterations in Raman spectra according to our previous study (Li et al., 2017) and was used in this study for Raman spectral acquisition.

To evaluate the transient and selective affinity response of each bacterial strain, the carbonaceous substance mixtures were prepared by mixing two carbonaceous substances, succinate/glucose for *A. baylyi* and acetate/glucose for *P. fluorescence* as well as *E. coli*; their final concentration was 10-mM:10-mM and 5-mM:10-mM, respectively. Bacterial cells were exposed to carbonaceous mixtures for 30 min at 30°C, followed by the same centrifugation and washing protocol as described above.

### Chemotaxis Capillary Assay

To evidence bacterial chemotactic propensity toward each carbonaceous substance, the conventional capillary assay was performed, as previously described (Adler, 1973; Li et al., 2017). The capillary tube (internal diameter of 0.2 mm and length of 10 cm) was first plunged into the carbonaceous substance stock solution for 5 min until the liquid was drawn up to approximately 1 cm in the tube. The tube was then inserted into the bacterial suspension for 30 min at 30°C. In order to measure the carbonaceous substance affinity of different bacterial strains, quantitative polymerase chain reaction (qPCR) was used to quantify the 16S rRNA copy numbers of chemotactic bacteria on each capillary in triplicates. The 1-cm exterior from the open end of the capillary tube was plunged into qPCR buffer as the DNA template. The 10 μL qPCR buffer contained 1 μL of primer 341F (5′-CCTACGGGNGGCWGCAG-3′), 1 μL of primer 802R (5′-TACNVGGGTATCTAATCC-3′), 3 μL of molecular water and 5 μL of iTaq^TM^ Universal SYBR^®^ Green Supermix (BioRad, United States). The thermo cycling program was: initial denaturation at 94°C for 3 min; 34 amplification cycles of 94°C for 45 s, 52°C for 45 s, 72°C for 45 s, and fluorescence data acquisition at 80°C for 15 s. The qPCR standard curves were obtained with serial dilutions of quantified plasmid DNA containing the fragment of 16S rRNA genes.

### Raman Spectral Acquisition

An InVia confocal micro-Raman system (Renishaw, Gloucestershire, United Kingdom) equipped with a 100 mW 785 nm excitation laser was employed for sample detection and Raman spectral acquisition, with a charged couple detector (CCD) and an attached microscope (Leica Microsystems, Milton Keynes, United Kingdom). The Raman system was calibrated using a Renishaw silicon calibration source before sample analysis. Ten microliter of washed cells were positioned onto a glass slide covered by aluminum foil and air-dried before Raman spectral acquisition (Cui et al., 2016). Twenty spectra per sample were randomly acquired using the 50 μm spectrometer entrance slit combined with a 1200 lines per mm diffraction grating (1 cm^–1^ spatial resolution), ×50 objective (0.75 numerical aperture; approximately 1 μm spatial resolution), 50% laser power (13 mW at samples), 10 s acquisition time and one accumulation within a spectral range from 500 to 2000 cm^–1^.

### Computational Analysis

Unless specifically stated otherwise, Raman spectra were pre-processed using baseline correction and vector normalization prior to principal component analysis (PCA) and linear discriminant analysis (LDA) using the IRootLab toolbox based on Matlab (version R2013b, MathWorks, United States) (Trevisan et al., 2013). PCA was carried out to visualize the natural variance within the dataset and reduce the dimensionality of the multivariate data (Trevisan et al., 2013). The separation of individual spectral categories from negative control and pure carbonaceous substance classes was measured by exporting PCA-derived data. To attain inter-class separation and minimize intra-class differences, LDA was employed to extract inter-category discriminating features (Martin et al., 2007; Butler et al., 2015). Post-exposure to different concentrations of carbonaceous substances, the dispersion of individual Raman spectra to that of negative controls (carbonaceous substance concentration = 0 mM) and pure carbonaceous substance was calculated based on principal component (PC)1 and PC2, and visualized as dispersion indicator (*D*_*I*_) score plots (Li et al., 2017). In a *D*_*I*_ score plot, the increasing *D*_*I*_ value between two categorizes is proportional to dissimilarity (Ami et al., 2013; Li et al., 2017).

## Results and Discussion

### Bacterial Chemotaxis Toward Carbonaceous Substance *via* Capillary Assay

The chemotaxis capillary assay of organic carbonaceous substance proved the different chemotactic sensitivities and binding affinities of *A. baylyi*, *P. fluorescence*, and *E. coli* toward glucose, acetate, succinate, and salicylate, respectively. From the results of the capillary assay, *A. baylyi* exhibited a highly sensitive chemotactic response toward glucose and succinate. The accumulation of *A. baylyi* 16S rRNA genes was 6.38 × 10^7^ and 6.75 × 10^7^ copies/capillary for glucose and succinate, respectively, significantly higher than those in acetate and salicylate treatments [see Electronic Supporting Information (ESI) Supplementary Figure S1]. In carbonaceous substance capillary assays of *P. fluorescence*, glucose was a strong chemo-attractant, achieving 2.13 × 10^7^ copies/capillary 16S rRNA genes (see ESI Supplementary Figure S1). *P. fluorescence* cells could also chemotactically sense acetate (1.18 × 10^7^ copies/capillary) or succinate (7.54 × 10^6^ copies/capillary), whereas only a small number of *P. fluorescence* cells accumulated in the salicylate capillary (1.29 × 10^6^ copies/capillary), indicating the weak chemotaxis of *P. fluorescence* toward salicylate. The chemotactic behavior of *E. coli* was entirely different from that of *A. baylyi* and *P. fluorescence*, consistent with previous studies (Singh and Arora, 2001; Kirby, 2009; Sourjik and Wingreen, 2012; Sampedro et al., 2015). *E. coli* showed weak chemotaxis-related affinity to succinate, and the 16S rRNA copy numbers post-exposure to the succinate were approximately 50 or 100-fold lower than that of *A. baylyi* or *P. fluorescence* (see ESI Supplementary Figure S1). Alternatively, the highest accumulation was achieved when post-exposure to acetate (1.57 × 10^6^ copies/capillary), whereas the accumulation was only 2.26 copies/capillary 16S rRNA genes in salicylate assay, showing the extremely weak salicylate attraction to *E. coli*.

### Raman Spectral Characterization of Bacterial Transient Affinity to Carbonaceous Substances

**FIGURE 1:**
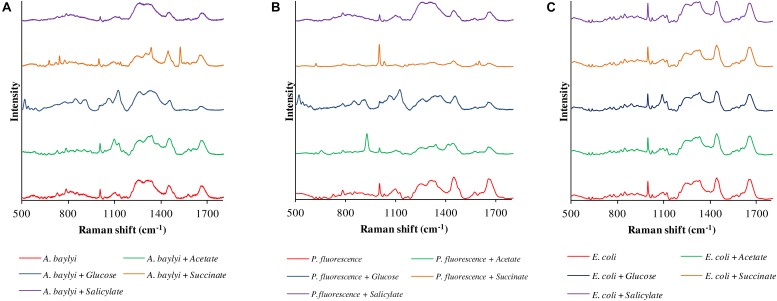
Raman spectra of *A. baylyi* **(A)**, *P. fluorescence* **(B)** and *E. coli* **(C)**, pre- and post-exposure to 10 mM acetate (green), glucose (blue), succinate (brown), and salicylate (purple). Twenty Raman spectra were randomly obtained per treatment.

**FIGURE 2:**
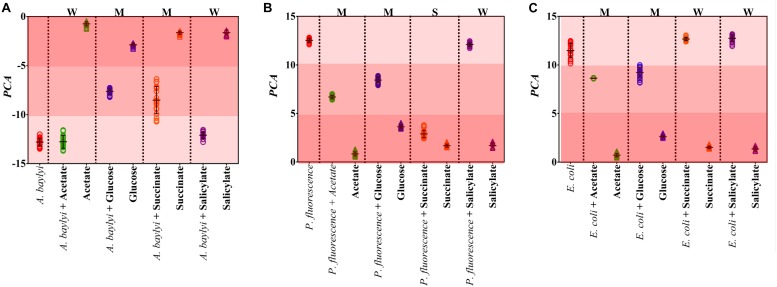
PCA score plots of *A. baylyi* **(A)**, *P. fluorescence* **(B)** and *E. coli* **(C)** post-exposure to different organic carbonaceous substances including acetate (green), glucose (blue), succinate (orange), and salicylate (purple). In each plot, color gradients illustrate the level of bacterial binding affinity toward the four carbonaceous substances. Light-red indicates weak affinity (W), medium-red represents moderate affinity (M), and dark-red refers to strong affinity (S). Twenty Raman spectra were randomly obtained per treatment.

Raman spectral alterations of the three bacterial strains post-exposure to four carbonaceous substances profile their transient binding affinity and cellular accumulation (Figure 1). Figure 2 further illustrates the PCA differentiation of the transient affinity and accumulation in *A. baylyi*, *P. fluorescence*, and *E. coli* for each organic carbonaceous substance. The three different color gradients of light-, medium-, and dark-red in PCA score plots represent weak, moderate, and strong levels of bacterial binding affinity toward different organic carbonaceous substances, respectively. After transient exposure (30 min) to the four carbon sources, distinct Raman spectra were identified for *A. baylyi*. Table 1 lists the predominant peaks in the Raman spectra of the pure *A. baylyi*, including 723 and 777 cm^–1^ (nucleic acids) (De Gelder et al., 2007), 1002 cm^–1^ (phenylalanine) (Li et al., 2017), 1238 and 1311 cm^–1^ (amino acids) (De Gelder et al., 2007), 1441 cm^–1^ (glycine) (Cui et al., 2013) and 1663 cm^–1^ (protein) (Li et al., 2017). Post-exposure to acetate or salicylate, the Raman spectra of *A. baylyi* remain unchanged, and the PCA of Raman spectra are indistinctive in comparison to the pure strains (Figures 1A, 2A). The PCA score plot indicates the obvious Raman alterations post-exposure to succinate (Figure 2A). The specific bands at 738, 1332, and 1446 cm^–1^ observed in the Raman spectra are attributed to C-H wag, sodium deformation and C-H bend in succinate, confirming the transient affinity and accumulation of succinate molecules in *A. baylyi* cells (Figure 1A) (Frost and Kloprogge, 2000; Dhanya et al., 2011). The PCA category comprising *A. baylyi* post-exposure to glucose is close to pure glucose, significantly different from the group of pure *A. baylyi* cells (Figure 2A). In Figure 1A, the glucose affinity and accumulation of *A. baylyi* contributes to the Raman spectral alterations, especially at 1338 cm^–1^ (-CH_2_ deformation), 1379 cm^–1^ (C-H bending and O-H bending) and 1454 cm^–1^ (-CH_2_ bending) (Vasko et al., 1972; Araujo-Andrade et al., 2005). These results are consistent with those of capillary assays, highlighting that *A. baylyi* has moderate chemotactic accumulation of succinate and glucose. It is worth noting that, compared to salicylate, the Raman spectral PCA categorization of *A. baylyi* post-exposure to acetate is closer to pure bacterial cells, whereas the capillary assays showed the opposite result that acetate attraction to *A. baylyi* was weaker than salicylate (see ESI Supplementary Figures S1, S2). The difference between the two approaches implies that the conventional capillary assay only evaluates the movement and accumulation of bacterial cells toward capillary, different from the detection of chemotaxis-driven accumulation and utilization of organic carbonaceous substances on/inside bacterial cells *via* Raman microspectroscopy. In addition, the cost of this Raman spectra acquisition was approximately $0.065 per sample (ESI Supplementary Table S1), showing its cost-effectiveness and feasibility for future practices. It hints at the applicability of employing Raman microspectroscopy to distinguish the vibrational fingerprints of organic carbonaceous substances on bacteria and further characterize the mechanisms of bacterial chemotaxis in complex environment.

**TABLE 1:** Assignments of Raman bands of three bacteria strains pre- and post-exposure to four carbonaceous substances.

	**Band (cm^–1^)**	**Tentative bands assignment**	***A. baylyi***					***E. coli***					***P. fluorescence***				
			**NC**	**Glu**	**Suc**	**Ace**	**Sal**	**NC**	**Glu**	**Suc**	**Ace**	**Sal**	**NC**	**Glu**	**Suc**	**Ace**	**Sal**
Bacteria	723	Nucleic acids	•	•	•	•	•	•	•	•	•	•	•	•	•	•	•
	777	Nucleic acids	•	•	•	•	•	•	•	•	•	•	•	•	•	•	•
	1002	Phenylalanine	•	•	•	•	•	•	•	•	•	•	•	•	•	•	•
	1238	Amino acids	•	•	•	•	•	•	•	•	•	•	•	•	•	•	•
	1311	Amino acids	•	•	•	•	•	•	•	•	•	•	•	•	•	•	•
	1441	Glycine	•	•	•	•	•	•	•	•	•	•	•	•	•	•	•
	1663	Protein	•	•	•	•	•	•	•	•	•	•	•	•	•	•	•

Glucose	838	C-H deformation	-	•	-	-	-	-	-	-	-	-	-	•	-	-	-
	914	C-H and C-O-H deformation	-	•	-	-	-	-	-	-	-	-	-	•	-	-	-
	1054	C-H deformation	-	-	-	-	-	-	-	-	-	-	-	•	-	-	-
	1116	C-H and C-O-H deformation	-	•	-	-	-	-	-	-	-	-	-	•	-	-	-
	1125	C-H and C-O-H deformation	-	-	-	-	-	-	-	-	-	-	-	•	-	-	-
	1338	-CH_2_ deformation	-	•	-	-	-	-	•	-	-	-	-	•	-	-	-
	1379	C-H bend and O-H bend	-	•	-	-	-	-	-	-	-	-	-	-	-	-	-
	1454	-CH_2_ deformation	-	•	-	-	-	-	-	-	-	-	-	-	-	-	-

Succinate	738	C-H wag	-	-	•	-	-	-	-	-	-	-	-	-	-	-	-
	939	C-C symmetric stretch	-	-	•	-	-	-	-	-	-	-	-	-	-	-	-
	1332	Sodium deformation	-	-	•	-	-	-	-	-	-	-	-	-	-	-	-
	1446	C-H bend	-	-	•	-	-	-	-	-	-	-	-	-	-	-	-

Acetate	652	-COO deformation	-	-	-	-	-	-	-	-	-	-	-	-	-	•	-
	928	C-C stretch	-	-	-	-	-	-	-	-	-	-	-	-	-	•	-
	1350	Metal deformation	-	-	-	-	-	-	-	-	•	-	-	-	-	•	-

*•, •, •, and •: Peak identified in corresponding Raman spectra of pure bacterial cells, glucose, succinate and acetate, respectively. -: no remarkable Raman peak is observed.*

Owing to the close genomic and proteomic relationship to the order *Pseudomonadales* between *A. baylyi* and *P. fluorescence*, the Raman spectra of these two bacterial strains are similar (Barbe et al., 2004; Young et al., 2005; Vaneechoutte et al., 2006). However, the transient response of *P. fluorescence* toward four carbonaceous substances is different from *A. baylyi*. Significant alterations are found in *P. fluorescence* post-exposure to acetate and glucose (Table 1). Post-exposure to glucose (Figure 1B), Raman band of 1338 cm^–1^ attributed to -CH_2_ deformation is dominant. Additionally, three glucose peaks are detected, occurring at 1125 cm^–1^ (C-H and C-O-H deformations), 1054 cm^–1^ (C-H deformation), and 914 cm^–1^ (C-H and C-O-H deformations) (Vasko et al., 1972; Araujo-Andrade et al., 2005). The remarkable difference in Raman spectral PCA score plots of *P. fluorescence* between pre- and post-exposure to glucose also proves the high glucose binding affinity of this bacterial strain (Figure 2B). In acetate treatment, the specific 1350 cm^–1^ band is attributed to sodium deformation (Figure 1B), (Frost and Kloprogge, 2000) and the bands 928 cm^–1^ (C-C stretch) and 652 cm^–1^ (-COO deformation) are also apparent (Figure 1B) (Bickley et al., 1990). Figure 2B illustrates the close distance of Raman spectral PCA groups between pure acetate and *P. fluorescence* post-exposure to acetate, demonstrating the moderate acetate affinity and accumulation. In contrast, no specific Raman spectral biomarker was observed in *P. fluorescence* post-exposure to salicylate and the PCA score plots suggest a weak attraction of salicylate (Figures 1B, 2B).

Similar to the carbonaceous substance affinity and accumulation in *P. fluorescence*, *E. coli* exhibits positive affinity toward glucose and acetate. Some Raman spectral peaks of pure *E. coli* are identical to those of *A. baylyi* and *P. fluorescence*, including 1002 cm^–1^ (symmetric ring breathing of phenylalanine) (Wang et al., 2011; Li et al., 2017), 1238 and 1331 cm^–1^ (amino acid) (De Gelder et al., 2007), 1441 cm^–1^ (-CH_2_ deformation in glycine) and 1663 cm^–1^ (C=C stretching of bacterial proteins) (Figure 1C) (De Gelder et al., 2007; Li et al., 2017). The strong glucose affinity of *E. coli* is highlighted in the PCA score plots, and the -CH_2_ deformation Raman band at 1338 cm^–1^ indicates the accumulation of glucose molecules on/inside *E. coli* cells (Figure 1C and Table 1). Post-exposure to acetate, PCA scores of *E. coli* are close to that of pure acetate, demonstrating a strong acetate affinity (Figure 2C). In succinate or salicylate treatments, no Raman spectral alteration was found for *E. coli*, a consequence of weak affinity and accumulation of succinate or salicylate on *E. coli*, similar to the results in capillary assay.

To quantify the binding affinity and accumulation of different organic carbonaceous substances on/inside bacterial cells *via* Raman spectra, Dispersion indicator (*D*_*I*_) is employed in this study to represent the dissimilarity between different treatments. Post-exposure to glucose, all the bacterial strains (*A. baylyi*, *P. fluorescence*, and *E. coli*) exhibit positive affinity. The *D*_*I*_ values of *A. baylyi*, *P. fluorescence*, and *E. coli* are 0.51, 0.72, and 0.22, respectively (see ESI Supplementary Figure S2). The results indicate moderate to strong binding affinity and cellular accumulation of glucose by all the strains. The Raman spectral *D*_*I*_ values are 0.36 and 0.43 for *A. baylyi* and *P. fluorescence* post-exposure to succinate, respectively, both higher than that of *E. coli* (see ESI Supplementary Figure S2). It suggests weak bacterial attraction of succinate toward *E. coli*. Post-exposure to acetate, the *D*_*I*_ values of *P. fluorescence* and *E. coli* are both higher than 0.5, indicating their strong acetate affinity. In salicylate treatment, all the *D*_*I*_ values of *A. baylyi*, *P. fluorescence*, and *E. coli* are all lower than 0.30 and hint at weak salicylate affinity.

**FIGURE 3:**
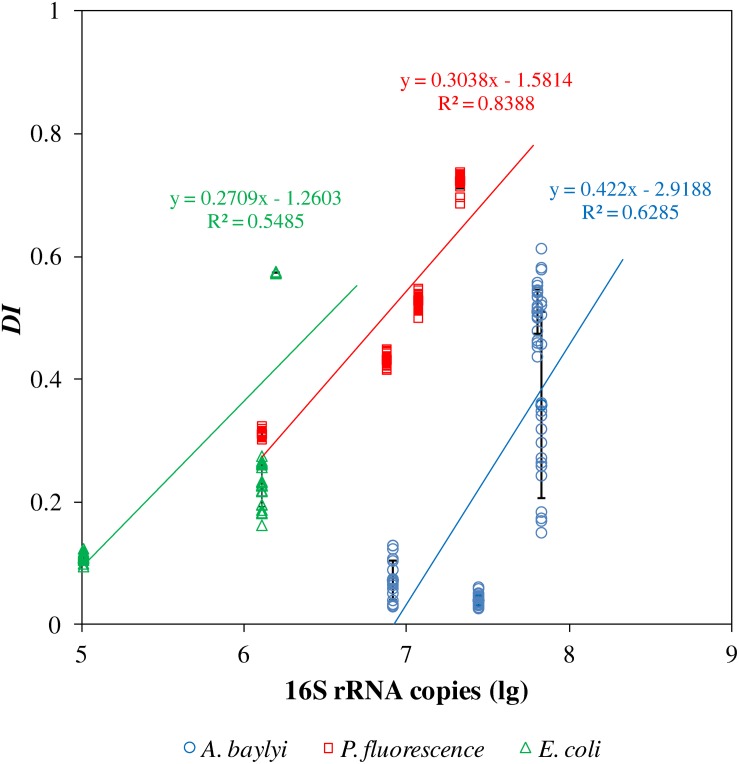
Semi-log linear regression of 16S rRNA copies per capillary and dispersion indicators (*D*_*I*_) of *A. baylyi*, *P. fluorescence* and *E. coli* post-exposure to different organic carbonaceous substances. R^2^ indicates the consistency of regression of *D*_*I*_ and 16S rRNA copies per capillary.

Raman microspectroscopy and capillary assay are different approaches to evaluate bacterial chemotaxis-driven affinity toward organic carbonaceous substances, using the distinct chemotactic indicators of *D*_*I*_ and 16S rRNA copies per capillary, respectively. The capillary assay quantifies the chemotaxis-driven movement and accumulation of bacterial strains to carbonaceous substances, whereas Raman spectral alterations illustrate the vibrational fingerprints of carbonaceous substances on/inside bacterial cells caused by binding affinity and accumulation (Li et al., 2017). We therefore generated a semi-log linear regression relationship between *D*_*I*_ scores in Raman spectral alterations and 16S rRNA copies in the capillary assay (Figure 3). The semi-log regression coefficient follows the order: *A. baylyi* (0.422) > *P. fluorescence* (0.308) > *E. coli* (0.271), indicating stronger chemotactic sensing and accumulation in *A. baylyi* and *P. fluorescence* than in *E. coli*. The R^2^ coefficient of determination is 0.5485 for *E. coli*, 0.6285 for *A. baylyi* and 0.8388 for *P. fluorescence*, showing good agreements between *D*_*I*_ and 16S rRNA copies/capillary, acquired from Raman spectral alteration and capillary assay, respectively. These results validate the *D*_*I*_ model in predicting bacterial chemotactic behavior, and *D*_*I*_ is a feasible indicator to quantify carbonaceous substance chemotaxis-related affinity and accumulation on/inside bacterial cells.

### Dose-Responsive Affinity and Accumulation of Carbonaceous Substances

**FIGURE 4:**
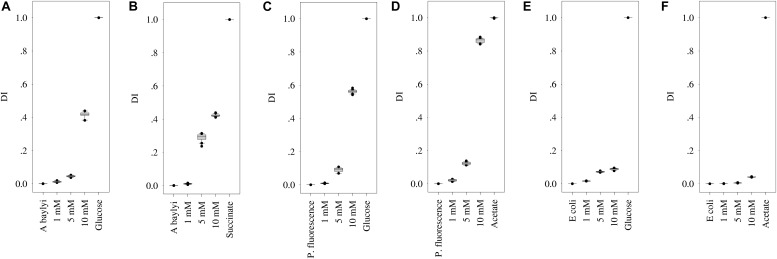
*D*_*I*_ score plots of bacterial Raman spectra after transient exposure to different concentrations of organic carbonaceous substances. **(A,B)** for *D*_*I*_ of *A. baylyi* cells post-exposure to glucose and succinate; **(C,D)** for *D*_*I*_ of *P. fluorescence* post-exposure to glucose and acetate; **(E,F)** for *D*_*I*_ of *E. coli* post-exposure to glucose and acetate. Twenty Raman spectra were randomly obtained per treatment.

The strong Raman spectral alterations of *A. baylyi*, *P. fluorescence*, and *E. coli* after transient exposure to carbonaceous substances allow one to quantify the binding affinity and cellular accumulation of organic carbonaceous molecules on/inside bacterial cells. The targeted carbonaceous substances are succinate and glucose for *A. baylyi*, and acetate and glucose for *P. fluorescence* and *E. coli*. Glucose had moderate to strong affinity and accumulation in the cells of all three strains. Thus, the intensity of characteristic peaks increased with glucose concentrations in all the treatments. The band at 1338 cm^–1^ (methylene deformation) was found in all the Raman spectra of *A. baylyi* post-exposure to glucose (see ESI Supplementary Figure S3). The Raman band of C-H and C-O-H deformation at 1116 cm^–1^ was positively correlated to the concentration of glucose in *A. baylyi* and *P. fluorescence*. Raman bands at 914 and 838 cm^–1^ bands assigned to C-H deformation were found in *A. baylyi* or *P. fluorescence* cells after transient exposure to 10-mM glucose. The *D*_*I*_ score plots (Figures 4A,C,E) illustrate a significant discrimination between the groups of *A. baylyi*, *P. fluorescence*, or *E. coli* post-exposure to 1, 5, or 10 mM glucose. For all the three strains, the group of 1 mM glucose was significantly segregated from the negative control (0 mM glucose). The *D*_*I*_ of *E. coli* post-exposure to 5 mM glucose was 0.073, of no difference with the 10 mM glucose treatment (*p* > 0.05). The results indicate a sensitive response of *E. coli* toward glucose, but the affinity and accumulation was weak at higher concentrations. In contrast, the *D*_*I*_ values of *A. baylyi* or *P. fluorescence* post-exposure to glucose constantly increased with glucose concentrations (Figures 4A,C), owing to the increasing glucose molecules accumulated on or inside *A. baylyi* or *P. fluorescence* cells. As a classic chemo-attractant, glucose-binding affinity to MCPs is well-documented (Hazelbauer et al., 1969; Singh and Arora, 2001; Weert et al., 2002). Bacterial chemotaxis is a significant step for binding affinity and cellular accumulation of glucose molecules. MCPs mediate the sensitivity of chemotaxis-driven affinity *via* a two-component regulatory system comprising a sensor kinase CheA and a response regulator CheY (Weert et al., 2002; Sourjik, 2004). As the transmembrane signal transducers triggered by the concentrations of extracellular glucose, MCPs are responsible for glucose recognition and accumulation on bacterial cells (Aizawa et al., 2000; Weert et al., 2002). Increasing Raman spectral alterations are therefore measurable at higher concentration of glucose and quantified by *D*_*I*_ scores.

For *A. baylyi* cells, we also observed the high binding affinity and cellular accumulation of succinate *via* Raman spectral alterations. The increasing concentrations of succinate raised the sodium deformation band at 1332 cm^–1^, and the Raman band of C-C symmetric stretch at 939 cm^–1^ was detectable when succinate concentration was >1 mM (see ESI Supplementary Figure S3). The 738 cm^–1^ band in *A. baylyi* post-exposure to 10 mM succinate was assigned to C-H wag. The *D*_*I*_ score plot illustrates that the group of *A. baylyi* post-exposure to 1 mM succinate were separated from negative control group (0 mM), confirming their highly sensitive affinity and accumulation of succinate (Figure 4B). Additionally, the *D*_*I*_ value of *A. baylyi* post-exposure to 5 mM succinate reached 0.29, significantly higher than that in 5 mM glucose treatment. It is reported that *A. baylyi* cells preferentially sense and accumulate straight chain dicarboxylic carbonaceous substances like succinate (Baumann et al., 1968; Parke et al., 2001). Therefore, succinate exhibits a higher attraction to *A. baylyi* especially at higher concentrations.

Post-exposed to acetate, Raman spectral alterations were detectable at 928 cm^–1^ (C-C stretch) in *P. fluorescence* and *E. coli*. The Raman bands at 652 cm^–1^ (sodium deformation) and 1350 cm^–1^ (carboxylic deformation) dominated the spectra of *P. fluorescence* post-exposure to 5 or 10 mM acetate (ESI Supplementary Figure S3). Figure 4D illustrates the increasing *D*_*I*_ values with acetate concentrations, from 0.021 (1 mM) to 0.862 (10 mM). It indicates the strong response and binding affinity of *P. fluorescence* to acetate. The *D*_*I*_ value of *P. fluorescence* post-exposure 10 mM acetate was much higher than that in 10 mM glucose (Figure 4D), although there was no significant difference between the categories of 5 mM acetate (0.09) and glucose (0.12). In addition, the *D*_*I*_ group of *P. fluorescence* in 1 mM acetate treatment was entirely discriminated from that of the negative control (Figure 4D), but not for *E. coli* (Figure 4F). Only 10 mM acetate exhibits obvious attraction to *E. coli* from the *D*_*I*_ score plot. The results suggest that *P. fluorescence* could recognize and accumulate 1 mM acetate, but the response sensitivity of *E. coli* toward acetate was 5 mM. Some previous studies have reported the strong acetate binding affinity in *P. fluorescence*, and the weak sensing and accumulation of acetate molecules in *E. coli* is attributed to the prolonged physiological adaptation in mineral medium (Paliy et al., 2003; Paliy and Gunasekera, 2007). Due to the low concentration of bivalent iron in mineral medium, the synthesis of enzymes is insufficient for acetate accumulation and uptake in *E. coli*.

### Selective Affinity and Accumulation Toward Carbonaceous Substance Mixtures

**FIGURE 5:**
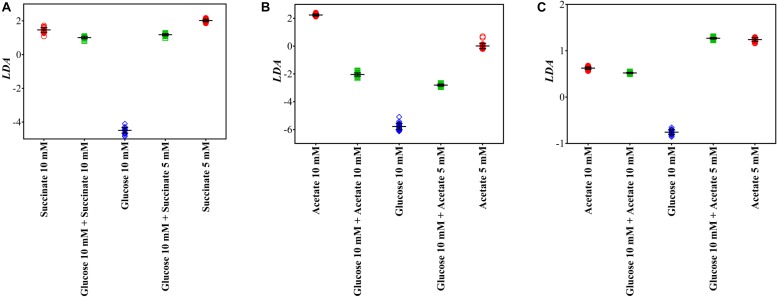
LDA score plots of *A. baylyi*, *P. fluorescence*, and *E. coli* after transient exposure to individual and multiple carbonaceous substances. **(A)** *A. baylyi* post-exposure to 10 mM succinate, 5 mM succinate, 10 mM glucose, and glucose-succinate (10 mM + 5 mM and 10 mM + 10 mM); **(B)** *P. fluorescence* post-exposure to 10 mM acetate, 5 mM acetate, 10 mM glucose, and glucose-acetate (10 mM + 5 mM and 10 mM + 10 mM); **(C)** *E. coli* post-exposure to 10 mM acetate, 5 mM acetate, 10 mM glucose, and glucose-acetate (10 mM + 5 mM and 10 mM + 10 mM).

Different to conventional capillary assay, which evaluates the movement and accumulation of bacterial cells toward carbonaceous substances, Raman microspectroscopy characterizes the chemotaxis-driven affinity, accumulation and utilization of organic carbonaceous substances on/inside bacterial cells *via* biospectral fingerprints. It allows one to distinguish which carbonaceous substance is selectively sensed and accumulated by bacterial cells and to discriminate the bacterial competitive affinity toward the mixtures of various carbonaceous substances. Figure 5 illustrates the LDA score plots of bacterial Raman spectra after transient exposure to individual and multiple carbonaceous substances, successfully evaluating the bacterial selective affinity and accumulation of individual carbonaceous substance in complicated nutrient environments.

Post-exposure to the glucose-succinate mixture (10 mM + 5 mM and 10 mM + 10 mM), the LDA categorization of *A. baylyi* cells is close to the groups of the same succinate concentration but markedly separated from the group of 10 mM glucose (Figure 5A). The difference in LDA position between LDA scores of multiple and individual carbonaceous substance treatments illustrates that only a small proportion of glucose molecules are sensed and accumulated on *A. baylyi* cells in the presence of both glucose and succinate (Figure 5A). Despite the decreasing concentration of succinate from 10 to 5 mM in the mixture, *A. baylyi* retains high binding affinity and accumulation of succinate. Namely, the molar ratio of succinate to glucose does not affect bacterial selectivity and the majority of *A. baylyi* cells have stronger binding affinity toward succinate. This result is consistent with the *D*_*I*_ values of *A. baylyi* post-exposure to individual glucose or succinate. *A. baylyi* cells show noticeable affinity toward 5 mM succinate (Figure 4B), whereas the significant attraction of glucose is only detectable at 10 mM (Figure 4A). It hints a stronger succinate binding affinity than glucose, and succinate is selectively sensed and accumulated in *A. baylyi* in succinate-glucose regardless of the occurrence of glucose. Although the MCPs of *A. baylyi* can recognize and bind glucose molecules, they are reported to have stronger binding affinity toward succinate (Juni, 1978; Young et al., 2005; Wang et al., 2013). The higher succinate binding affinity up-regulates the chemotaxis signal transduction and promotes the activities of CheA autophosphorylation (Garrity and Ordal, 1997). As a result, the movement of *A. baylyi* cells is more positively promoted toward succinate than glucose, leading to the increasing and predominant affinity and accumulation of succinate.

From the LDA plots of *P. fluorescence* post-exposure to acetate or/and glucose (Figure 5B), LDA categorization of the glucose-acetate mixture groups, either 10 mM + 5 mM or 10 mM + 10 mM treatment, are both located with the same distance between the groups of individual acetate and glucose. Meanwhile, higher acetate concentration (10 mM) in the mixture does not significantly increase the selective affinity and accumulation of *P. fluorescence* toward acetate (Figure 5B). The results demonstrate a neutral competitive affinity and accumulation toward acetate and glucose by *P. fluorescence*. Compared to the results in *D*_*I*_ score plots (Figures 4C,D) that the chemotactic behaviors of *P. fluorescence* are similar post-exposure to 5 mM acetate or glucose but significantly stronger for 10 mM acetate than 10 mM glucose, the neutral selectivity suggests different chemoreceptors for acetate and glucose in *P. fluorescence*. Previous studies report that *Pseudomonas* sp. have diverse MCP-like proteins and are strongly chemotactic toward a variety of attractants (Ramos, 2004). The chemoreceptor for acetate in *Pseudomonas* strains is McpS, a cluster II ligand binding region for TCA cycle intermediates (Lacal et al., 2011; Pineda-Molina et al., 2012; Sampedro et al., 2015). This acetate chemotaxis protein is different from that for glucose, but following the same downstream signal transduction pathway in *P. fluorescence* (Weert et al., 2002; Sampedro et al., 2015). Thus, post-exposure to acetate-glucose mixture, different MCPs exhibit limited effects on the chemotaxis signal transduction of other chemo-attractants.

Post-exposure to glucose or acetate, Raman spectral alterations show almost the same binding affinity and accumulation of these two carbonaceous molecules on *E. coli* cells from *D*_*I*_ score plots (Figures 4E,F). We therefore speculate a similar LDA categorization of *E. coli* in glucose-acetate mixture comparing to that of *P. fluorescence*. However, Raman spectra LDA score plots illustrate that groups of *E. coli* treated with two acetate-glucose mixtures are remarkably close to those in individual acetate treatments (5 and 10 mM, Figure 5C), indicating a strong selection toward acetate rather than glucose. Additionally, the concentration of acetate does not affect the selective affinity, and there is a significant difference in Raman spectral LDA score plot for the two groups between acetate-glucose mixtures with 5 and 10 mM acetate. It is obvious that, post-exposure to glucose-acetate mixtures, *E. coli* exhibits a more sensitive response to acetate than glucose. The difference between individual and multiple exposure illustrates the distinct selective behaviors of *E. coli* cells in a complex carbonaceous substance circumstance. Namely, although *E. coli* has moderate chemotaxis-driven affinity and accumulation of glucose, similar as acetate, the glucose sensing is strongly inhibited by the presence of acetate. In *E. coli* cells, strong binding affinity decreases CheA autophosphorylation and weakens the chemotaxis signal transduction (Borkovich et al., 1989). In contrast, acetate can activate CheY phosphorylation *via* acetylation and promote *E. coli* chemotaxis (Dailey and Berg, 1993). Although acetylation is not essential for chemotaxis, it can offset the negative effects of CheA autophosphorylation in acetate-glucose mixture, and as a result, encourages acetate affinity and accumulation. Thus, increasing Raman spectral biomarkers of acetate on *E. coli* cells are identified post-exposure to acetate-glucose mixture, and our work for the first time successfully observes the significantly selective chemotaxis-driven affinity and accumulation between two carbonaceous substances with similar chemotactic behavior.

Raman spectra can measure bacterial binding affinity to individual carbonaceous substance and can also identify which molecule is sensed and accumulated on bacterial cells in a carbonaceous substance mixture. Our results suggest that Raman microspectroscopy is an effective tool in discriminating biospectral fingerprints and revealing the mechanisms of bacterial selective affinity and accumulation in complex nutrient environments. To our knowledge, and compare with the results of capillary assay, we believe the selective affinity and accumulation of bacteria toward the mixture of carbonaceous substances is related to the chemotactic regulatory mechanism. Comparing the different behaviors of the three bacterial strains toward individual or carbonaceous substance mixtures, we find three different types of chemo-attractants with distinct roles in MCPs recognition and signal transduction. When the signal transduction is not regulated by MCPs recognition and affinity, *P. fluorescence* toward glucose and acetate (neutral-chemo-attractant) as an example, there is no significant difference between individual and multiple carbonaceous substances. In the case of up-regulating chemotaxis signal transduction by MCPs recognition and affinity (succinate as active-chemo-attractant for *A. baylyi*), bacterial cells have strong selectivity and preferentially swim toward and accumulate active-chemo-attractant post-exposure to multiple carbonaceous substances. Conversely, if MCPs recognition and affinity down-regulates the signal transduction, like *E. coli* toward glucose as an inactive-chemo-attractant, bacterial binding affinity will prefer other types of chemo-attractants and less inactive-chemo-attractant is sensed. Bacterial chemotaxis is a complex biological behavior in seeking out and accessing chemical stimuli, and both chemo-attractant sensing and signal transduction play important roles in chemotactic behavior. As the chemoreceptor protein, MCPs are able to recognize the periplasmic cognate ligands and deliver the adaptive response through methylation and demethylation (Ferrández et al., 2002). The signal is subsequently transmitted to regulate CheA kinase, CheW scaffolding protein and CheY signal transmission protein to control flagellar motor response (Wadhams and Armitage, 2004; Mukherjee et al., 2016). Some mechanisms of chemotaxis signal transduction pathways have been discussed for several bacterial strains (Borkovich et al., 1989; Garrity and Ordal, 1997; Sampedro et al., 2015; Mukherjee et al., 2016). CheA autophosphorylation and CheY phosphorylation regulate flagella rotation, leading to the change in swimming direction or a tumble (Welch et al., 1993; Toker and Macnab, 1997; Wadhams and Armitage, 2004). Our results from Raman spectral alterations and multivariate analysis suggest diverse mechanisms of bacterial chemotactic selectivity, varying across bacterial species and carbonaceous substances. Post-exposure to multiple carbonaceous substances, the chemo-affinity and chemotaxis signal transduction both play remarkable roles in the bacterial chemotactic behavior (Sampedro et al., 2015). Raman microspectroscopy allows one to elucidate the possible mechanisms of cell-molecule interaction *via* distinguishing specific molecular biomarkers, especially in a complex nutrient environment.

## Conclusion

In the present study, we applied Raman microspectroscopy to investigate the transient binding affinity and accumulation of organic carbonaceous substances on/inside bacterial cells after extremely short-term exposure (30 min). To the best of our knowledge, this is the first study successfully discriminating the vibrational fingerprints of carbonaceous substances on/inside bacterial cells and revealing the different mechanisms of selective accumulation and utilization *via* Raman spectra coupled with multivariate analysis. After transient exposure to carbonaceous substance, Raman spectral alterations can visualize the molecular biomarkers in biospectra and quantify the selective affinity and accumulation of carbonaceous molecules. Furthermore, the selective behaviors uncovered by Raman microspectroscopy provide deeper insights into the complicated association of MCPs recognition and chemotaxis signal transduction in different bacterial strains. The selective affinity and accumulation are important for bacterial access to adequate nutrients in their natural habitats, in which multiple carbonaceous substances co-exist. Our work suggests that Raman microspectroscopy is a robust and sensitive tool, with great potential in diagnosing bacteria-molecule interaction *in situ* at single cell level. Although the present study only proves the concept, it is feasible for non-destructive detection of living cells by real-time Raman spectroscopy in further study. Discriminating bacterial selective access to carbonaceous molecules in real world scenario, particularly for those uncultivable microbes, also helps our deeper understanding on microbial functions in natural environment.

## Data Availability Statement

The datasets analyzed in this manuscript are not publicly available. Requests to access the datasets should be directed to the corresponding author.

## Author contributions

DZ and FM designed the study. HL conducted the experiments, interpreted the data, and contributed to the data analysis. HL and DZ wrote the manuscript. FM, KJ, and DZ revised the manuscript.

## Conflict of Interest

The authors declare that the research was conducted in the absence of any commercial or financial relationships that could be construed as a potential conflict of interest.
