# Supplementary material for: Interrogating the Transient Selectivity of Bacterial Chemotaxis-Driven Affinity and Accumulation of Carbonaceous Substances via Raman Microspectroscopy
Source: Front Microbiol. 2019 Oct 4;10:2215. doi: 10.3389/fmicb.2019.02215 (PMC6787638; doi:10.3389/fmicb.2019.02215)
Supplement: Supplementary file 1 [file Table_1.docx]

**Electronic Supporting Information**

**Interrogating the transient selectivity of bacterial chemotaxis-driven affinity and accumulation of carbonaceous substances via Raman microspectroscopy**

Hanbing Li^a,b^, Francis L Martin^c^, Kevin C. Jones^b^, Dayi Zhang^d,*^

^a^ State Key Laboratory of Pollution Control and Resource Reuse, School of the Environment, Nanjing University, Nanjing, Jiangsu, 210023, China

^b^ Lancaster Environment Centre, Lancaster University, Lancaster LA1 4YQ, UK

^c^ School of Pharmacy and Biomedical Sciences, University of Central Lancashire, Preston PR1 2HE, UK

^d^ School of Environment, Tsinghua University, Beijing, 100084, PR China

***Corresponding author**

Dr Dayi Zhang

School of Environment, Tsinghua University, Beijing, 100084, PR China

Lancaster Environment Centre, Lancaster University, Lancaster LA1 4YQ, UK

Tel.: +86(0)62773232; Fax: +86(0)62785687; Email: [zhangdayi@tsinghua.org.cn](mailto:zhangdayi@tsinghua.org.cn)

No. of Pages = 4

No. of Tables = 1

No. of Figures = 3

**Table S1. Cost evaluation for Raman spectra acquisition**

| **Materials or consumables** | **Price ($/unit)** | **Sample per unit** | **Cost ($/sample)** | |  |
| --- | --- | --- | --- | --- | --- |
| **Materials for cultivation** | 2.0 | 100 | 0.02 | |  |
| **Slides and aluminium foil** | 0.075 | 5 | 0.015 | |  |
| **785-nm laser source** | 3,000 | 100,000 | 0.03 | |  |
| **Total** | | | | 0.065 | |

Note: Cost are from Sigma-Aldrich (USA) for materials or HORIBA (Japan) for laser source.

**Figure S1.** Chemotaxis of *A. baylyi, P. fluorescence* and *E. coli* towards glucose, acetate, succinate and salicylate *via* the capillary assay.

**Figure S2.** Dispersion indicator (*D_I_*) of bacterial Raman spectra post-exposure to glucose, acetate, succinate and salicylate.


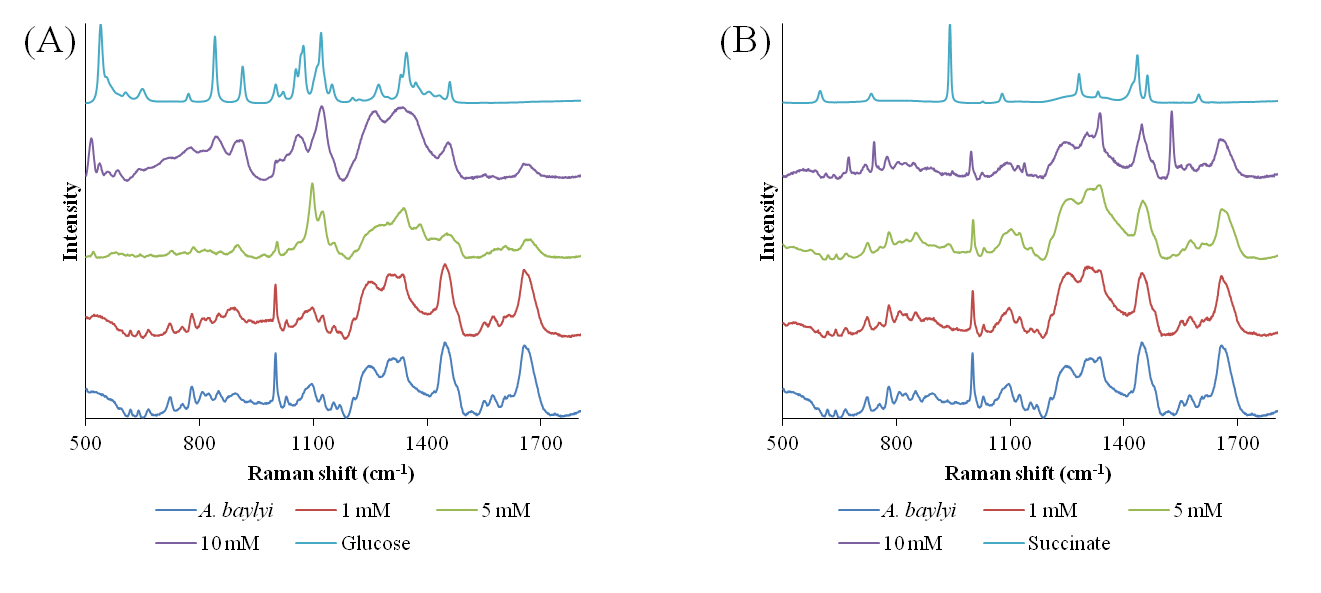


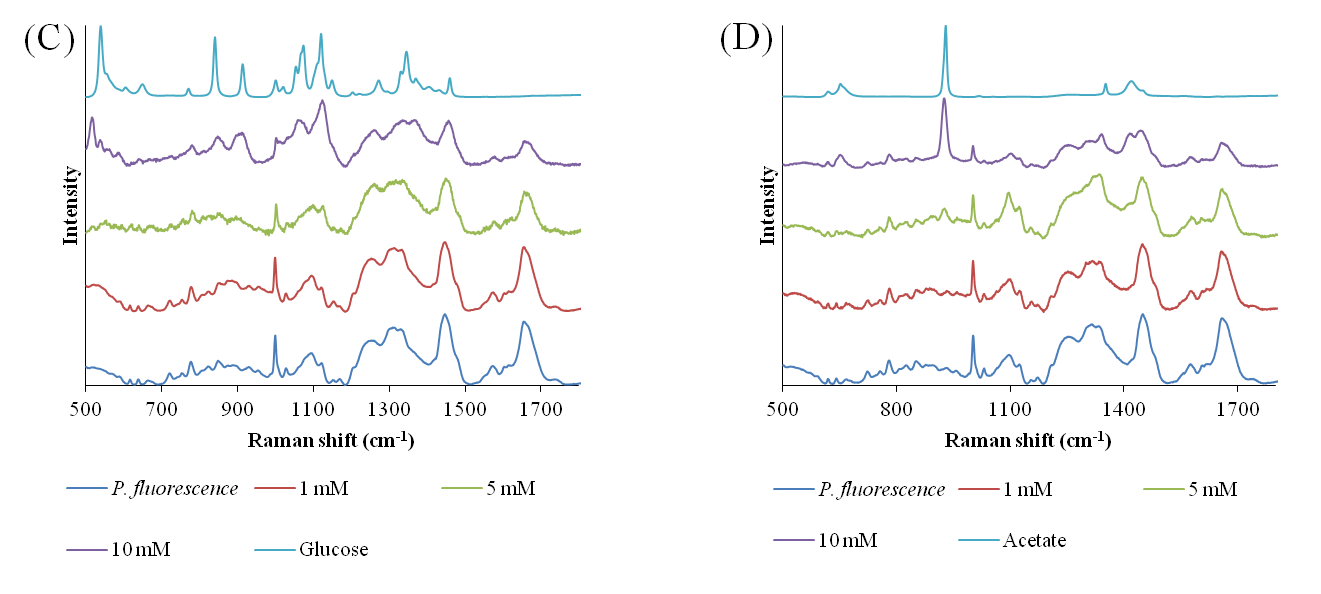

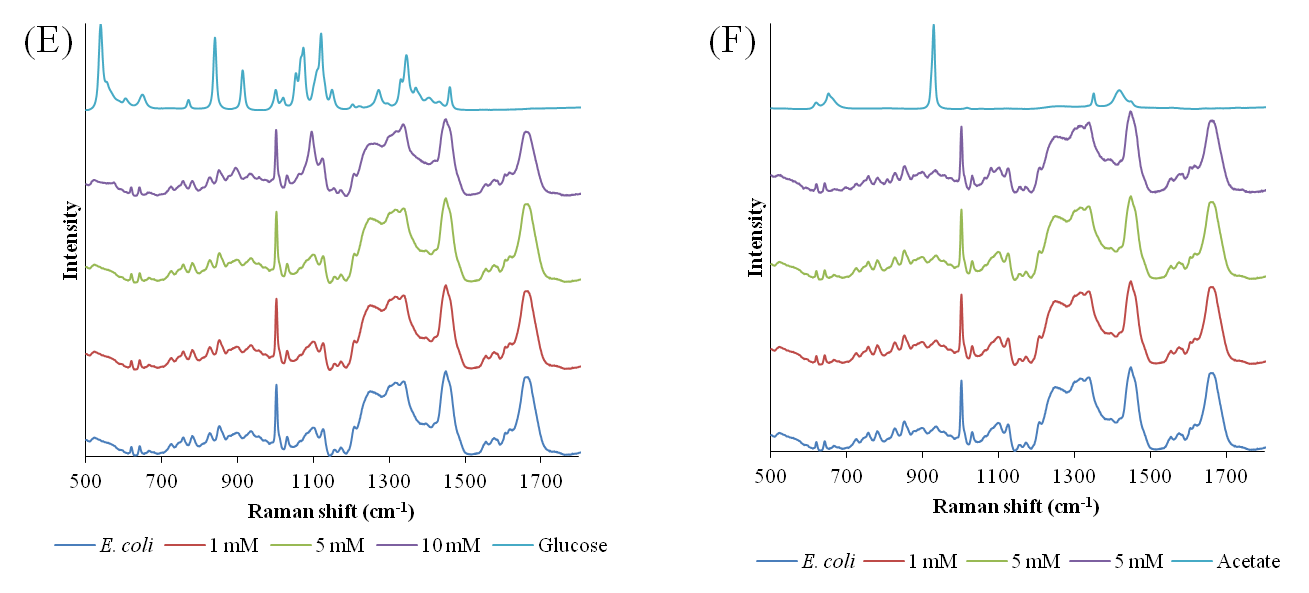


**Figure S3.** Bacterial Raman spectra post-exposure to different concentrations of organic carbonaceous substances. (A) and (B): *A. baylyi* post-exposure to glucose and succinate; (C) and (D): *P. fluorescence* post-exposure to glucose and acetate; (E) and (F): *E. coli* post-exposure to glucose and acetate. Twenty Raman spectra were randomly obtained per treatment.
